# Effects of an Educational Hybrid Physical Education Program on Physical Fitness, Body Composition and Sedentary and Physical Activity Times in Adolescents: The Seneb’s Enigma

**DOI:** 10.3389/fpsyg.2020.629335

**Published:** 2021-01-12

**Authors:** David Melero-Cañas, Vicente Morales-Baños, David Manzano-Sánchez, Dani Navarro-Ardoy, Alfonso Valero-Valenzuela

**Affiliations:** ^1^Department of Physical Activity and Sport, CEI Campus Mare Nostrum, University of Murcia, Murcia, Spain; ^2^Department of Physical Education and Sports, School of Sport Sciences, University of Granada, Granada, Spain

**Keywords:** physical health, body mass index, afterschool period, model-based learning, sedentary behavior

## Abstract

Physical activity (PA), body composition and sedentary behavior may affect the health of children. Therefore, this study examined the effect of an educational hybrid physical education (PE) program on physical fitness (PF), body composition and sedentary and PA times in adolescents. A 9-month group-randomized controlled trial was conducted in 150 participants (age: 14.63 ± 1.38 years) allocated into the control group (CG, *n* = 37) and experimental group (EG, *n* = 113). Cardiorespiratory fitness, speed, strength, agility, flexibility and body mass index (BMI) were assessed through previously validated field tests. Sedentary time, PA at school and afterschool were evaluated with the Youth Activity Profile-Spain questionnaire. Significant differences were observed concerning to the CG in APA-weekend (*p* = 0.044), speed-agility (*p* = 0.005) and agility (*p* = 0.008). Regarding the intervention, cardiorespiratory fitness (*p* = 0.000), speed-agility (*p* = 0.000), strength (*p* = 0.000), flexibility (*p* = 0.000), agility (*p* = 0.000), PA in school (*p* = 0.011), APA-weekday (*p* = 0.001), APA-weekend (*p* = 0.000), APA-week (*p* = 0.000), and sedentary time (*p* = 0.000) increased significantly in the EG. The use of a hybrid program based on teaching personal and social responsibility and gamification strategies produced enhancements in cardiorespiratory fitness, agility, speed, APA-weekdays and APA-weekends, reducing the sedentary time.

## Introduction

The Constitution of the World Health Organization (WHO) defines health as “a state of complete physical, mental and social well-being and not merely the absence of disease or infirmity” (World Health Organization, 1946). They assert that both physical and mental well-being are human rights, enabling a life without limitation or restriction. Consistent with this notion, non-communicable diseases are global public health issues that lead to premature death and disability (Global Burder of Disease 2015 Mortality and Causes of Death Collaborators, 2016). Specifically, the prevalence of physical inactivity, which is considered a key modifiable driver of childhood obesity, has reached alarming levels among European youth (Gómez et al., 2020), with the consequent problems on social, economic and personal levels (Martines et al., 2019; Nittari et al., 2019).

Among the many risk factors, cardiovascular disease and development and progression of a sedentary lifestyle are now recognized as leading contributors to poor cardiovascular health (Poortvliet et al., 2003). Low cardiorespiratory fitness in childhood and adolescence has been associated with increased risk for death and disability later in life (Neovius et al., 2008; Högström et al., 2016; Henriksson et al., 2019). In this sense, being overweight can contribute to cardiometabolic diseases, such as diabetes or hypertension (Reilly and Kelly, 2011). Reilly et al. considered that more than 77% of overweight children were found to be overweight or obese as adults. For this reason, it is important to know if the relationship among physical activity (PA), body composition and sedentary behavior could be relevant factors in the health of children.

Overall, regular exercise and PA are associated with remarkable widespread health benefits, such as lower blood pressure or higher insulin sensitivity (Nystoriak and Bhatnagar, 2018), directly related to obesity and a sedentary lifestyle (Poortvliet et al., 2003; Nystoriak and Bhatnagar, 2018; Rodríguez-Ayllon et al., 2019). Furthermore, the afterschool period (e.g., 4 PM–8:30 PM) has been recognized as a relevant and feasible time period with a decrease in PA and an increase in sedentary time among children, because they are not restricted by school schedules and have the opportunity to engage in their sedentary pastimes (Riddoch et al., 2004). Additionally, several studies have observed a negative relationship between those factors (sedentarism and PA) and body max index (BMI) in children (Gao et al., 2011), specifically higher in the afterschool period (Morgan et al., 2007; Colley et al., 2013). The BMI is a factor that can influence the PA level since children that are overweight engage in less PA (Colley et al., 2013; Blanco et al., 2020), with consequent problems, such as neurotrophic growth factors (Mora-González et al., 2019a) and conduct problems, since less PA is associated with a less balanced diet and is positively associated with alcohol consumption in teenagers of both sexes (Grao-Cruces et al., 2015). The European Youth Heart Study determined that devoting 60 min or more to moderate-vigorous PA daily is associated with a healthier cardiovascular fitness level in adolescents (Poortvliet et al., 2003) and better postural control (García-Soidán et al., 2020) and cognitive performance (Ishihara et al., 2020). In addition, there is a close relationship between physical fitness (PF) and PA (Martínez-Vizcaíno and Sánchez-López, 2008). A high level of PF can help to improve the optimal health status and prevent a wide variety of disease morbidities and mortalities (Metter et al., 2002; Kodama et al., 2009). On the other hand, a high level of PF will imply a good physiological response to musculoskeletal, cardiorespiratory, hemato-circulatory, endocrine-metabolic and psychoneurological levels. It will be inversely proportional whether PF is low (Ortega et al., 2008b).

Educational centers have been identified as the leading places for promoting PA and health, especially during physical education (PE) classes (Sallis and McKenzie, 1991; Kahan and McKenzie, 2015). In this sense, it is important to promote PA during PE and morning recess in order to increase moderate and vigorous physical activity (MVPA), especially in children with overweight/obesity, because they report less PA in all daily segments, including the educational context (Pope et al., 2020). The recent recommendations of WHO for children and adolescents aged 5–17 are they should do at least an average of 60 min per day of MVPA, incorporating those that strengthen muscle and bone, at least 3 days a week (World Health Organization, 2020). Besides that, Gao et al. (2011) identified that children with overweight/obesity participated less in MVPA in PE classes than children of healthy weight. According to gender, it seems that there are differences between boys and girls, with higher values of MVPA in boys, regardless of whether they are measured during the week or on weekends (Aibar et al., 2014). Other studies have not identified differences according to gender in teenagers (Manzano-Sánchez and Valero-Valenzuela, 2018) or children (Svedenkrans et al., 2020).

However, it is necessary to say that there is a tendency of reduction in PA increase with age between childhood and adolescence (Arundell et al., 2013), and the absence of MVPA is important to improve health in children. Sedentary behavior also has a significant influence, leading to poorer health outcomes (Colley et al., 2013). For this reason, it is important measure PF, sedentary time, and PA. To measure PA, some studies used pedometers to measure PA in children and teenagers (Tudor-Locke et al., 2006; Isensee et al., 2018; Fang et al., 2020). It has some advantages, such as the cost, but they can not have the ability to measure different intensities (like MVPA). This is why accelerometry is one of the most reliable instruments to measure the PA of people (Pope et al., 2020). One of the main purposes of PE classes is to improve some variables to increase the adherence to PA in children and teenagers. Some studies that have used model-based learning can improve motivation, autonomy and competence, and these variables have a significant relation with MVPA (Manzano-Sánchez and Valero-Valenzuela, 2018). Recently, the Teaching Personal and Social Responsibility (TPSR) model has demonstrated improvements in these variables (Martínez-Vizcaíno and Sánchez-López, 2008; Manzano-Sánchez and Valero-Valenzuela, 2019; Manzano-Sánchez et al., 2019]. The latter was also demonstrated with the application of a gamification intervention (Pérez-López et al., 2017a,b) that was implemented on a long-term basis in different educational levels (primary and secondary education), social status and educational backgrounds (Fernández-Río et al., 2020).

For these reasons, the objective of the present study was to verify whether a hybrid educational program in PE classes based on TPSR and gamification techniques can increase the parameters of PF and PA; apart from that, it can reduce body composition and sedentary behavior. Furthermore, gender and age differences will be considered in order to check the results.

## Materials and Methods

### Study Design

**FIGURE 1:**
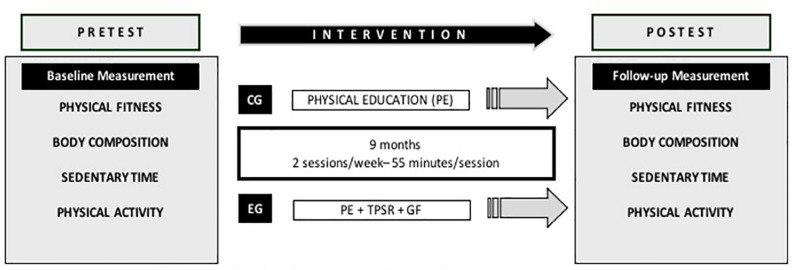
Variables and timeline intervention.

A group-randomized controlled trial (Montero and León, 2007) was carried out from September 2018 to June 2019. The intervention program lasted for 9 months (Figure 1) in two secondary schools assigned to the control group (CG) or experimental group (EG). Sociodemographic and cultural characteristics were similar. Participants aged between 13 and 15 years had to be enrolled in the second or third year of Compulsory Secondary Education at the beginning of the intervention in one of the two secondary schools selected. The contents were selected according to the current education laws (BOE, 2014). Before and after the intervention, the students were required to carry out the tests in two different sessions. Informed consents (participation in the study, confidential data treatment and session recording) were requested from the students and their parents. The study was approved by the Ethics Committee of the University of Murcia (2871/2020).

### Exclusion Criteria and Participants

Participation was proposed to all students enrolled in one of the courses. Participants did not have to present any partial or chronic injury or disease that would prevent them from performing any of the physical and cognitive tests, participating normally in PE lessons or not having been diagnosed as a student with specific needs for educational support.

**FIGURE 2:**
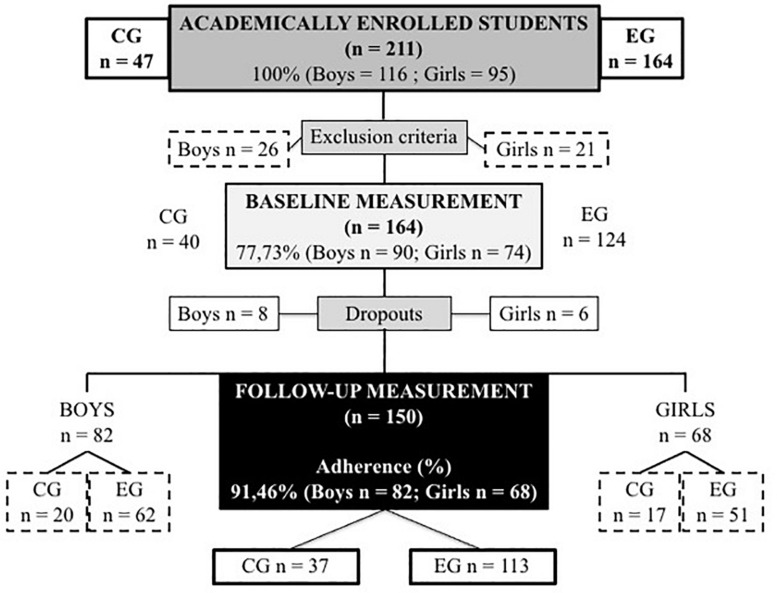
The flowchart of the participants.

Initially, 211 adolescents began the intervention (Figure 2), with 164 (age: 14.63 ± 1.38 years; 77.73% of the total) who finally formed part of the experience (90 boys and 74 girls) allocated to the CG (*n* = 40) and EG (*n* = 124).

However, a total of 150 participants completed the study, specifically 91.46% of the total number of students who started it, 37 in the CG and 113 in the EG. The reasons why 14 students did not finish the study, three from the CG (2 boys and 1 girl) and 11 from the EG (6 boys and 5 girls) were due to school absenteeism, missing more than 20% of PE lessons (*n* = 6) and discomfort during the performance of any of the physical or cognitive tests in the post-test (*n* = 8). Table 1 shows the characteristics of the adolescents who started and finished, as well as the variables.

**TABLE 1:** Initial data of the participants and variables.

	Participants (*n* = 150)		EG (*n* = 113)		CG (*n* = 37)		*p*
	Mean	SE	Mean	SE	Mean	SE	
Age (years)	14.33	1.18	14.52	1.24	13.76	0.68	
Height (cm)	156.2	4.82	154.6	3.54	154.03	2.94	
Weight (kg)	48.4	3.24	50.20	3.91	49.46	3.02	
BMI	19.89	6.63	21.46	4.11	21.04	4.05	0.589
PAIS	2.86	0.85	2.86	0.88	2.90	0.73	0.801
APA (weekday)	2.82	0.87	2.71	0.87	3.17	0.79	0.005**
APA (weekend)	2.49	0.97	2.35	0.87	2.95	0.97	0.001**
APA (week)	2.69	0.78	2.57	0.77	3.08	0.71	0.000**
ST	2.66	0.57	2.72	0.57	2.46	0.51	0.017**
CF	4.28	2.28	3.99	2.27	5.08	2.11	0.011**
SPD-AGI	13.16	1.26	13.27	1.29	12.78	1.10	0.041**
Strength	1.52	0.36	1.50	0.37	1.57	0.34	0.325
Flex (average)	3.65	7.64	3.51	7.63	4.38	7.60	0.548
Agility	14.67	2.31	14.93	2.18	13.69	2.17	0.003**

*SE, standard error; EG, experimental group; CG, control group; BMI, body mass index; PAIS, physical activity in school; APA, afterschool physical activity; ST, sedentary time; CF, cardiovascular fitness; SPD-AGI, speed/agility; Flex, Flexibility.*

Each group (CG and EG) received two PE lessons per week, lasting 55 min. Whereas the EG participated in a PE program based fundamentally on the hybridization of TPSR and gamification strategies, taking into account game-based learning, the CG used traditional learning methods characterized by non-integration and lack of transfer of learning outside of school. Moreover, the teacher of the CG had no experience in active methodologies like those mentioned above. Experimental and control teachers were filmed by an external observer in order to verify the fidelity of implementation of TPSR and gamification techniques in 10 random sessions (Valero-Valenzuela et al., 2020). The instruments used were the same following a similar evaluation structure and also both experimental and control teachers evaluated their own intervention (self-evaluation). The observer, person trained in the application and evaluation of this type of pedagogical model, analyzed both methodological behaviors and evaluated the frequency that teachers used the hybridization learning models, ranging from 1 to 4 (from never to always). This expert was trained in thanks to a researcher with more than 5 years of experience in this methodology beforehand to check the quality of their record-keeping by calculating the inter-observer and intra-observer reliability concordance using Cohen’s kappa coefficient (Cohen, 1988), of which a mean value of more than 0.70 was obtained. Inter reliability was carried out between the observer and the researcher, while the intra-observer was carried out analyzing two different moments over one week, guaranteeing an agreement greater than 80%.

The check list instrument (Table 2) and the Tool for Assessing Responsibility-Based Education (TARE) were used to identify the gamification and responsibility elements, respectively (Wright and Craig, 2011).

**TABLE 2:** Fidelity Implementation Instrument (gamification elements).

	Control group			Experimental group		
	SE	EO	Mean SE/EO	SE	EO	Mean SE/EO
1. Setting (ST). Uses background music and/or decorates the place with respect to the narrative of intervention.	2	2	2	4	3	3.5
2. Mechanics (MC). Grants rewards and provides feedback on the accomplishment of the challenges.	1	1	1	4	4	4
3. Dynamics (DN). Introduces a narrative thread into the session. Generates curiosity.	2	1	1.5	3	3	3
4. Components (CO). Generates missions, realms (groups), roles/status, badges, rankings and markers.	1	1	1	4	4	4
5. Leadership (L). Allows students to lead or be in charge of a group.	2	2	2	4	4	4
6. Working in small groups (WSG). Organizes activities and tasks in small groups (4–6 students).	3	2	2.5	4	4	4
7. Task exceeded by the whole group (TEG). Encourages all teammates to succeed.	1	1	1	4	4	4
8. Setting challenges (SC). Tries the challenges that arise during the development of the sessions.	2	1	1.5	4	4	4
9. Groups stability over time (GST). Tries to maintain stability in the kingdoms/groups formed over time for several sessions.	1	1	1	4	4	4
10. Identification/characterization (ID/C). Characterizes students in a way that identifies them with a kingdom/group.	2	1	1.5	4	3	3.5
Total mean	1.69	1.30	1.49	3.92	3.76	3.84

### Procedure

#### TPSR Intervention Program

The sessions followed the Hellison‘s format (Hellison, 2011) but were modified to keep four of its five parts: (1) Initial greeting: the teacher interacted with the students to create bonds with them; (2) Sensitivity talk: the teacher presented the academic and value goals of the session, depending on the responsibility model level; (3) Activity plan: this was the greatest part of the practical lesson, where responsibility strategies were included in the different tasks; and (4) Group meeting and self-assessment: at the end of every session, teacher and students shared their perceptions with regard to individual and collective responsibilities and behaviors, as well as the teacher’s behavior, pointing their thumbs up (positive evaluation), to one side (medium) or down (negative evaluation).

#### Gamification Strategies

Regarding the gamification elements in the intervention program, the process of integrating game-design principles within varying educational experiences appear challenging, and currently, there are no practical guidelines for how to do coherently and efficiently (Dichev and Dicheva, 2017). Therefore, based on the three categories (dynamics, mechanics and components) mentioned by Werbach and Hunter (2014), the following elements were included as part of the gamificated context: (a) Powerful narrative: Seneb’s Enigma was designed as the common theme in order to discover a complete health, previously extinct; (b) Challenges: on each mythology, students had to reach two different activities, generally outside the school and each one included different difficulty levels to challenge the students individually and in groups; (c) Class climate: the focus was on performing the different tasks, such as helping group members, earning points, and earning badges, and not on outperforming others; (d) Immediate feedback: students knew in advance how to successfully perform each activity, the number of points awarded for each task, and the level effectively achieved through a social platform; (e) Badges for achievements: students could earn points (“healthy years”) to obtain several badges on each unit; and (f) Final status: depending on the mythologies overcome, students reach one of the three status (squires, Egyptian “melli” and bearers of Seneb).

Moreover, this intervention has taken in account the four key motivational elements (RAMP) of Marczewski (2013), that a gamified experience should incorporate itself: *Relatedness* or the desire to be connected to others in a social community; *Autonomy*, or the freedom in order to not be controlled or stifled; *Mastery*, or the process of becoming skilled at something feeling their skills are increasing in direct proportion to the level of challenge; *Purpose*, or the meaning of their actions. The reason why it is necessary the intervention.

#### Control Group Methodology

Direct instruction was the methodology used by the CG teacher based on content/skill development and teacher-centered decisions and without affective-social interaction with the students, causing automatisms in their learning (Metzler, 2005). Virtually every element was monitored and decided on by the teacher, including content selection, managerial control, task presentations, engagements patterns, instructional interaction, pacing and task progression. The teacher decided when practice started and stopped remaining in full control of the class. Students did not have to make decisions besides participation in the different tasks. His principal goal was paying attention to the result but not to the learning process. The format of each session was divided into three non-connected parts: (1) warm-up: students got ready for the class performing predesigned tasks (i.e., joint mobility); (2) main part: students performed a predesigned set of tasks to improve the selected skills (i.e., badminton hitting drills); and (3) cool down: students performed lighter tasks to get ready for the next class, focusing on stretching their muscles and following the instructions of the teacher (Metzler, 2005).

### Instruments and Measurements

#### Physical Fitness

The PF assessment protocol was used in the previously published European HELENA study (Healthy Lifestyle in Europe by Nutrition in Adolescence^1^) (Ruiz et al., 2006; Ortega et al., 2008a, 2009). The PF tests used have shown optimal validity and reliability in order to be applied in the adolescent population (Ortega et al., 2005; Castro-Pinero et al., 2010; Ardoy et al., 2011). Cardiorespiratory fitness was assessed by the 20 m shuttle run test; speed and agility were evaluated by the 4 × 10 m speed-agility test; lower body strength was measured by the standing broad jump; and low back flexibility was evaluated by the back saver sit-and-reach test and calculating the average of both legs. Additionally, the hexagon test was used to evaluate agility-coordination and dynamic balance (Beekhuizen et al., 2009).

#### Body Composition

The assessment of body composition was proposed and used in the HELENA study. The descriptions of the measurements made, the material used for this purpose and the reliability analysis have been previously published (Nagy et al., 2008). Body mass and height measurements were previously taken with the SECA-876 brand height rod and scale, and were used to calculate BMI (kg/m^2^).

#### Lifestyle Habits

The Youth Activity Profile-Spain (YAP-S) questionnaire (α = 0.73 and 0,79; pre- and post-test) was used to analyze the time spent practicing PA (in school and afterschool) and sedentary activities, such as watching television, playing video games or using mobile phones. The reliability was for the whole sample who finished the intervention. It has previously been validated (Sain-Maurice and Welk, 2015) and used in more intervention studies (González-Gross et al., 2003; Ardoy et al., 2010).

### Data Analysis

Initially, we carried out the validation of the instrument by analyzing its internal consistency, both in the pre-test and in the post-test of each of the variables, using Cronbach’s alpha test to assess reliability. Then, we carried out an exploratory analysis of the data using box-whisker diagrams and descriptive measurements, detecting that there were significantly different results between groups in the pre-test, so this was taken into account in the inferential analysis that was carried out. Afterschool physical activity (weekday, weekend and week), sedentary time, speed-agility, cardiorespiratory fitness and agility-coordination variables were significantly different in CG.

In a first analysis, a MANOVA of repeated measurements was carried out on the 12 variables obtained from the different PF tests and questionnaire, where we called the intra-subject factor “Time” (with two levels: pre-test and post-test) and we called the inter-subject factor “Group” (with two levels: control and experimental). Additionally, the inter-subject age factor and gender were added as covariates, since we found that this factor could have a significant effect on the measured variables. Additionally, the intervention effect size was estimated using the Cohen’s d (Cohen, 1988) with Hedges correlation for small sample sizes (Nakagawa and Cuthill, 2007). The effect size was considered small when it was 0.2–0.5, medium when it was 0.51–0.8 and large when it was greater than 0.8. The entire statistical analysis was performed with the Statistical Package for the Social Sciences (IBM SPSS 24.0), establishing the level of significance *p* < 0.05.

## Results

This section may be divided by subheadings. It should provide a concise and precise description of the experimental results, their interpretation and the experimental conclusions that can be drawn.

### Inferential Analysis

The MANOVA of repeated measurements at the multivariate level was the first step in the analysis. With regard to the inter-subject analysis, there was a significant difference in the Gender factor (Lambda of Wilks = 0.405; *F* = 16.436; *p* = 0.000) but not with the Age covariate (Lambda of Wilks = 0.870; *F* = 1.663; *p* = 0.082). It was also observed that there were significant differences in the intra-subject analysis between Time and Group interactions (Lambda of Wilks = 0.462; *F* = 12.993; *p* = 0.000). The fact that the Time factor (Lambda of Wilks = 0.860; *F* = 1.812; *p* = 0.052) was not significant did not mean that there were no differences between the pre-test and the post-test, since there were significant interactions with the Group factor. This indicates that there may be differences in the Time factor (i.e., between pre-test and post-test) depending on the group considered (differences for each group separately), as well as taking this factor into account (pre-test and post-test differences between the CG and EG).

In order to observe more specifically which variables showed significant differences, the univariate level was analyzed. Attention was paid to the variables with previously significant results. For the intra-subject factor, significant differences were obtained in Time and Group interactions for APA-weekend (*p* = 0.000), APA-week (*p* = 0.000), sedentary time (*p* = 0.002), BMI (*p* = 0.000), cardiorespiratory fitness (*p* = 0.000), speed-agility (*p* = 0.000), strength (*p* = 0.001), and agility (*p* = 0.000).

Since there were interactions between the Time and Group factors for many of the variables, we can analyze the differences between the CG and the EG for the pre-test and the post-test separately. Similarly, the variables in the pre-test and post-test should be compared separately for each group. Thus, Table 3 reflects the means and standard errors estimated for the participants with regard to the different variables measured in the pre-test and in the post-test, differentiating by group. In addition, the p-values obtained by comparing these estimated averages (using Bonferroni correction) are included.

**TABLE 3:** Intervention multivariate analysis (MANOVA).

Variable	Group	Pre-test		Post-test		Pre – post comparative	
		Mean	SE	Mean	SE	*p*-value	Dif. (SE)
PA in school	Experimental	2.86	0.88	3.04	0.78	0.011*	−0.18 (0.067)
	Control	2.90	0.73	3.24	0.86	0.005**	−0.34 (0.120)
	*p*-value + SE	0.956	0.167	0.247	0.152		
APA (weekday)	Experimental	2.71	0.87	3.04	0.91	0.001**	−0.33 (0.095)
	Control	3.17	0.79	3.16	0.85	0.912	0.01 (0.169)
	*p*-value + SE	0.006**	0.163	0.551	0.173		
APA (weekend)	Experimental	2.35	0.87	3.02	1.01	0.000**	0.67 (0.090)
	Control	2.95	0.97	2.65	0.82	0.020*	0.30 (0.161)
	*p*-value + SE	0.000**	0.169	0.044*	0.185		
APA (week)	Experimental	2.57	0.77	3.03	0.82	0.000**	−0.46 (0.074)
	Control	3.08	0.71	2.96	0.71	0.223	0.12 (0.132)
	*p*-value + SE	0.000**	0.141	0.560	0.151		
Sedentary time	Experimental	2.72	0.57	2.47	0.54	0.000**	0.25 (0.059)
	Control	2.46	0.51	2.64	0.58	0.203	−0.18 (0.105)
	*p*-value + SE	0.024*	0.110	0.238	0.107		
BMI	Experimental	21.46	4.11	21.50	4.05	0.521	−0.04 (0.093)
	Control	21.04	4.05	21.94	3.44	0.000*	−0.90 (0.166)
	*p*-value + SE	0.934	0.810	0.355	0.774		
CF	Experimental	3.99	2.27	5.04	2.20	0.000**	−1.05 (0.116)
	Control	5.08	2.11	4.70	2.19	0.066	0.38 (0.207)
	*p*-value + SE	0.001**	0.351	0.443	0.349		
SPD-AGI	Experimental	13.27	1.29	11.63	1.53	0.000**	1.64 (0.098)
	Control	12.78	1.10	12.38	1.21	0.043*	0.40 (0.174)
	*p*-value + SE	0.004**	0.198	0.005**	0.256		
Strength	Experimental	1.50	0.37	1.64	0.38	0.000**	−0.14 (0.015)
	Control	1.57	0.34	1.61	0.37	0.268	−0.04 (0.027)
	*p*-value + SE	0.110	0.056	0.723	0.058		
Flexibility (average)	Experimental	3.51	7.63	5.82	7.51	0.000**	−2.31 (0.232)
	Control	4.38	7.60	6.03	8.22	0.000**	−1.65 (0.412)
	*p*-value + SE	0.496	1.382	0.924	1.381		
Agility	Experimental	14.93	2.18	12.30	1.93	0.000**	2.63 (0.142)
	Control	13.69	2.17	13.30	2.60	0.203	0.39 (0.252)
	*p*-value + SE	0.003**	0.415	0.008**	0.394		

*p < 0.05; **p < 0.01; SE, standard error; PA, physical activity; APA, afterschool physical activity; BMI, body mass index, CF, cardiorespiratory fitness; SPD-AGI, speed/agility.

It is relevant to indicate that, in the pre-test, there were significant differences between groups in several variables of interest. However, it is relevant to remark that there were significant differences in the post-test with APA-weekend (*p* = 0.044), speed-agility (*p* = 0.005), and agility (*p* = 0.008). In addition, there were no significant enhancements with PA in school, APA-week, sedentary time and cardiorespiratory fitness. These last two variables are extremely relevant because they were worse in the CG with respect to the pre-test.

On the other hand, if it compares the effects of intervention, observing the results between the pre-test and post-test for each group, it can observe the same for the CG. There were significant differences in PA in school (*p* = 0.005), speed-agility (*p* = 0.043), flexibility (*p* = 0.000), BMI (*p* = 0.000), and APA-weekend (*p* = 0.020). However, the last two variables decreased concerning to the pre-test. Regarding the EG, the results increased significantly in all variables, except BMI, including cardiorespiratory fitness (*p* = 0.000), speed-agility (*p* = 0.000), strength (*p* = 0.000), flexibility (*p* = 0.000), agility (*p* = 0.000), PA in school (*p* = 0.011), APA-weekday (*p* = 0.001), APA-weekend (*p* = 0.000), APA-week (*p* = 0.000), and sedentary time (*p* = 0.000).

## Discussion

The main purpose of this study was to verify whether a hybrid educational program in PE classes based on TPSR and gamification can increase the parameters of PF and PA; on the other hand, it can reduce body composition and sedentary behavior.

The results observed in the present study indicate that the implementation of a hybrid program based on TPSR and gamification contributes significantly to the improvement of variables such as cardiorespiratory fitness, speed-agility, strength, flexibility and agility. Data supports the conclusions of González-Víllora et al. (2019), where combined benefits in the physical/motor, cognitive, affective and social domains have only been observed when merging different pedagogical models concerning to the intervention of an isolated pedagogical model. However, TPSR has never been hybridized with gamification strategies. The hybridizations of pedagogical models in the scientific literature with TPSR as the protagonist have been related to the improvement of psychosocial variables and personal and social development (Hastie and Buchanan, 2000; Menéndez-Santurio and Fernández-Río, 2016; Fernández-Río and Menéndez-Santurio, 2017). However, the variables related to health, physical-motor and cognitive aspects have never been analyzed through pedagogical hybridizations (González-Víllora et al., 2019), as our intervention has carried out, demonstrating that, despite reducing motor physical involvement in the early stages of applying the model, their levels of cardiorespiratory fitness, speed-agility, strength, flexibility and agility were improved. Apart from that, the application of gamification elements in isolated PE interventions has not only contributed to the increase in student motivation and commitment toward PA practice (Joo et al., 2019), but also to the improvement of cardiorespiratory capacity (Mora-González et al., 2019b, 2020) or healthy lifestyle habits (Monguillot-Hernándo et al., 2015; Pérez-López et al., 2017a).

That intervention also produced enhancements in PA in school, APA-weekday, APA-weekend, APA-week and sedentary time, as well as an increase in BMI in the group that did not receive it. These results would be related to those obtained in other studies in which they linked the decrease in PA levels to overweight parameters and neurotrophic growth factors problems (Mora-González et al., 2019a) as well as to close future morbidity and mortality disease (Metter et al., 2002; Kodama et al., 2009). Additionally, our results, largely obtained by activities carried out outside of school, would be in line with the studies proposed by Arundell et al. (2013), suggesting that afterschool period is an important time period to enhance PA levels and sedentary time in childhood and adolescence and reducing BMI (Gao et al., 2011). Accordingly to this study, our results indicated that BMI increased significantly in students who did not participate in the intervention and therefore did not perform extra physical activity. It may be the reason to indicate the BMI raised for CG. Other studies (Harris et al., 2009; Thivel et al., 2011; Guerra et al., 2013) have shown non-significantly improvements not only for the EG, but also for the CG (Thivel et al., 2011) in both normal weight and obese children, highlighting the structuring and durability of the intervention program and the insufficient “dose” of physical activity (Harris et al., 2009). A possible reason why PA in school has significantly improved in both group may be the PE sessions themselves, because are based on practice exercise regardless of the type of methodology used. For this reason, it will be so relevant to incorporate this educational program to current curricular requirements in order to promote PA during PE lessons (Pope et al., 2020) and afterschool (Arundell et al., 2013).

Some limitations in this study are that it may not be a sufficient and optimal representation of the population of their age. Consequently, this intervention study may consider the unequal distribution of the participants, with the CG being reduced compared to the EG. It could be convenient not to extend the post-intervention tests until dates close to the evaluations established by the educational centers due to possible experimental deaths. Moreover, the performance of PF tests was determined according to the time assigned in the timeline hours of each group, as a consequence of the curricular organization school.

Due to the time difficulty and number of trained and qualified personnel for the test days, both at the beginning and at the end of the intervention, the evaluators were not the same at both times. It could be a limitation, even though they were previously trained.

Another variable that could affect the reliability of the results is the activity carried out after school hours. The number of days per week they do training, the type of sport or predominant PA and its relationship with the intensity variables, volume and duration of effort should be considered in students who belong to sport teams.

Additionally, other limitation was the instrument used in order to measure the elements of gamification, because it is not a validated instrument and it has never been published. Future studies may consider the use of accelerometry, which would allow a more precise evaluation of daily PA.

## Conclusion

The results obtained with the intervention suggested that the use of a hybrid program based on TPSR and gamification strategies produced enhancements in cardiorespiratory fitness, agility and speed-agility. In addition, it improves the APA-weekdays and APA-weekends, reducing the sedentary time. On the other hand, students who did not receive an intervention based on a hybrid of these methodologies were linked to an increase in BMI.

The states and administrations for education should consider the inclusion of innovative programs and their hybridization as a tool to guarantee an optimal state of health in adolescents. Additionally, the afterschool period, concerning to overall PA (particularly during adolescence), may be crucial in this age group. Future studies involving larger sample sizes should confirm or contrast these preliminary findings.

## Data Availability Statement

The raw data supporting the conclusions of this article will be made available by the authors, without undue reservation.

## Ethics Statement

The studies involving human participants were reviewed and approved by Ethics Committee of the University of Murcia (2871/2020). Written informed consent to participate in this study was provided by the participants’ legal guardian/next of kin.

## Author Contributions

DM-C and AV-V: conceptualization. DM-C and DN-A: methodology and investigation. DM-C, VM-B, and DM-S: formal analysis. VM-B: resources. DM-C and DM-S: data curation. DM-C: writing—original draft preparation. DM-C and VM-B: writing—review and editing. AV-V: visualization. AV-V and D**N-**A: supervision. All authors have read and agreed to the published version of the manuscript.

## Conflict of Interest

The authors declare that the research was conducted in the absence of any commercial or financial relationships that could be construed as a potential conflict of interest.

## Footnotes

1 www.helenastudy.com
